# Epigenetic clocks for gestational age: statistical and study design considerations

**DOI:** 10.1186/s13148-017-0402-y

**Published:** 2017-09-15

**Authors:** Andrew J. Simpkin, Matthew Suderman, Laura D. Howe

**Affiliations:** MRC Integrative Epidemiology Unit at the University of Bristol, Population Health Sciences, Bristol Medical School, University of Bristol, Oakfield House, Oakfield Grove, Bristol, BS8 2BN UK

**Keywords:** Prediction, DNA methylation, Gestational age at delivery, Epigenetic clock

## Abstract

In this letter to the editor, we highlight some concerns with a recently published method to estimate gestational age at delivery from DNA methylation data. We conduct novel analyses to highlight the implications of different choices in study design and statistical methods for the prediction of phenotypes from methylation data.

## Letter to the editor

In two recent articles, Bohlin et al. [1] and Knight et al. [2] develop DNA methylation clocks for gestational age at delivery (GA) using cord and newborn blood. These follow from the clock of Horvath [3], which has been used to obtain a measure of age acceleration, the discrepancy between estimated and chronological age, calculated as the residual when regressing one on the other. Associations between age acceleration and a wide range of phenotypes have been observed, including all-cause mortality [4]. The newly developed GA clocks offer the similar possibility to assess determinants of GA acceleration (GAA) and associations with subsequent outcomes. A recent article in this journal used the GA clock developed by Knight et al. to examine associations with maternal and offspring characteristics [5].

We applied both GA clocks to cord blood DNA methylation data from 863 members of the ARIES cohort [6] in order to test associations of GAA with birth weight. We observed a strong positive correlation with the Bohlin GAA estimate (*R* = 0.20, *p* = 5.3 × 10^−9^) and a comparatively weak correlation with the Knight estimate (*R* = 0.055, *p* = 0.11).

To investigate this discrepancy, we asked how well both clocks estimated GA in ARIES. Although both were correlated with GA (Bohlin *R* = 0.65; Knight *R* = 0.37), these correlations are below those reported in their original publications (Bohlin *R* = 0.81; Knight *R* = 0.91). In the case of Bohlin, this is expected given that their training and testing sets were drawn from the same cohort study. This was not the case for Knight. The scatterplot of the Knight test results (Figure 1b of [2]) suggested that the strong correlation may have been driven by the inclusion of data for 183 preterm infants in the test set. We tested this hypothesis by adding these preterm infants to ARIES, increasing the percentage of preterm infants (GA < 37 weeks) from 2.8 to 19.8%. As expected, the correlation of the Knight GA estimate rose from *R* = 0.37 in ARIES alone to *R* = 0.89.

Given the much lower correlation of the Knight compared to the Bohlin estimate in ARIES, we asked if the difference could be due to differences between their training datasets: GA measured using ultrasound (Bohlin) versus last menstrual period (Knight), mean GA (Bohlin 39.9; Knight 36.9), training samples (1068 for Bohlin; 207 for Knight), 450K CpG sites (Bohlin) versus 27K CpG sites (Knight). Most of these predict that Bohlin will perform best in an average population like ARIES. We did, however, wonder if the Knight clock performance could have been improved had more training samples been used. Whereas the Knight training dataset consisted of six cohorts with 207 samples, the testing dataset consisted of > 1000 samples. We also wondered if the Knight clock consisting of nearly as many CpG sites (148) as training samples (207) might have suffered from overfitting.

To explore these concerns, we derived a new GA clock by fitting elastic nets implementing in the glmnet R package to the publicly available subset of the Knight testing and training data restricted to the same 27K CpG sites (total *n* = 400; GSE36642 *n* = 51; GSE62924 *n* = 38; GSE79056 *n* = 36; GSE80310 *n* = 24; GSE66459 *n* = 22; GSE69633 *n* = 46; VICS *n* = 183). The resulting clock included 193 CpG sites, and its correlation with GA in ARIES was quite similar to the Knight estimate (*R* = 0.56). Both the original Knight clock and our new clock were correlated with measured GA (*R* = 0.37) and with the Bohlin estimate at similar strength (*R* = 0.49). Both GAA estimates were also similar (R = 0.49) and were correlated with the Bohlin GAA estimate at similar strength (*R* = 0.35). However, the new GAA estimate was more strongly correlated with birth weight (*R* = 0.09; *p* = 0.008) compared to the Knight estimate (*R* = 0.055, *p* = 0.11).

Although our clock was less likely to suffer from overfitting (193 CpG sites from 400 samples), we still considered the ratio of CpG sites to training samples to be quite high. To investigate, we also generated clocks restricted to 50, 25, and 10 CpG sites. The resulting clocks were still strongly associated with GA (*R* = 0.4, 0.33, and 0.25, respectively) and with the default 193-CpG clock (*R* = 0.82, 0.72, and 0.61, respectively). GAA estimates were similarly strongly associated with the GAA estimate of the default clock (*R* = 0.79, 0.68, and 0.57, respectively) and with birth weight (*R* = 0.12, 0.12, and 0.11, respectively). These results show that 50 CpG sites was sufficient to produce GA and GAA estimates with optimal performance.

To conclude, care must be taken when deriving and testing molecular models. Test dataset characteristics should match the datasets where the model will be later applied. Training datasets should be as large as possible. Although it is necessary to reserve data for testing in order to assess performance, it would be useful if authors also published a model trained using *all* available data. In most cases, the model derived from the large dataset will be superior. Finally, model sizes should be restricted to reflect the size of the training data to avoid overfitting.
